# Contrast-enhanced ultrasound guided pleural biopsy improves diagnostic confidence for pleural based lesions: a 3-year prospective study

**DOI:** 10.1186/s12890-021-01583-7

**Published:** 2021-07-12

**Authors:** Wenwen Sun, Yiming Zhou, Cong Yang, Zhengwei Dong, ZheMin Zhang, Yin Wang, Lin Fan

**Affiliations:** 1 Department of Tuberculosis and Shanghai Key Lab of Tuberculosis, Shanghai Pulmonary Hospital, School of Medicine, Tongji University, Shanghai, 200092 China; 2 Department of Thoracic Surgery, Shanghai Pulmonary Hospital, School of Medicine, Tongji University, Shanghai, 200092 China; 3 Department of Ultrasound, Shanghai Pulmonary Hospital, School of Medicine, Tongji University, Shanghai, 20092 China; 4 Department of Pathology, Shanghai Pulmonary Hospital, School of Medicine, Tongji University, Shanghai, 200092 China; 5 Shanghai Pulmonary Hospital, School of Medicine, Tongji University, Shanghai, 200092 China

**Keywords:** Contrast-enhanced ultrasound, Ultrasound guided pleural biopsy, Pleural lesion, Diagnostic efficiency, Infectious pleural lesion, Malignant pleural lesion

## Abstract

**Objective:**

To evaluate the accuracy and safety of contrast-enhanced ultrasound (CEUS) guided biopsy in the diagnosis of radiologically determined pleural based lesions.

**Method:**

A prospective study was conducted on patients with radiologically determined pleural based lesions. Patients who met the inclusion criteria received pleural biopsy guided by CEUS to obtain specimens, followed by histomathological and microbiological examinations. After treatment and follow-up, surgical thoracoscopy was performed on cases with undefinite diagnosis.

**Result:**

A total of 460 patients were finally included. CEUS showed internal necrosis in 72.17% cases and obvious peripheral vessels in 55.43% cases, both of which were significantly higher than the conventional ultrasound imaged (*p* < 0.05). The diagnostic accuracy through CEUS guided biopsy sampling was 98.91% (455/460). The microbiological diagnostic yield achieved 71.88% (225/313) in infectious lesions. In 330 cases combined pleural effusion, CEUS guided biopsy increased the diagnostic yield from 60.30% (199 /330) to 98.36% (325 /330) in all cases (*p* < 0.05), from 15.56% (14/90) to 94.44% (85/90) in malignant lesions (*p* < 0.01) and from 77.08% (185/240) to 100% (240/240) in infectious lesions (*p* < 0.05). No serious adverse events occurred.

**Conclusion:**

CEUS guided biopsy provides a minimally invasive, effective and safe diagnostic biopsy method for pleural lesions.

*Clinical Trials Registration*: Chinese Clinical Trial Registry ChiCTR2000029749 (ChiCTR, www.chictr.org.cn).

## Introduction

Pleural lesion is a chest imaging manifestation of a variety of diseases, of which the diagnosis covers multidisciplinary areas regarding surgery, histopathology, microbiology and imaging, etc [1]. The best specimen for the diagnosis of pleural disease is pleural tissue rather than pleural effusion [1]. Direct sampling from the target lesion is required for microbiology and pathology examinations. Previous studies had shown that thoracoscopic pleural biopsy was considered to be the best method for the diagnosis of pleural effusion of unknown causes and was particularly useful in the diagnosis of malignant pleural disease with the diagnostic rate of 90–95% [2–4]. However, it is well known that thoracoscopic biopsy is an invasive procedure which may not be the first choice in source-limited countries, especially in areas with high TB burdens. Ultrasound-guided closed biopsy (USCB), as a minimally invasive method, was advocated as a first-line initial diagnostic procedure for undiagnosed pleural effusion, especially in areas with high TB burdens [5, 6]. However, conventional ultrasound could not better distinguish atelectasis from necrotizing lesions, which may leads to false negative biopsy outcomes and complications [6, 7]. Contrast-enhanced ultrasound (CEUS) is a technology developed on the basis of conventional ultrasound imaging, which can display real-time information of pathological microvascular and blood perfusion. The detection of tissue microcirculation after the injection of contrast agent can distinguish diseased tissue activity from necrotic area then provide a suitable site for biopsy. In addition, CEUS guided biopsy can conduct real-time guided and accurate targeted puncture biopsy, so as to avoid injury to adjacent organs and large blood vessels, which has the advantages of accurate guidance and fast access to high-quality specimens [8].

Previous study included 58 patients with pleural-based peripheral pulmonary lesions to evaluate the diagnostic value of CEUS guided biopsy and obtained an overall diagnostic rate of 98.3% [9]. Our previous studies also found the diagnostic efficiency of CEUS guided biopsy in superficial lymph nodes and pleural tuberculosis [10, 11]. Furthermore, we conducted a 3-year prospective clinical study to evaluate the value-added effect of CEUS-guided pleural biopsy in the diagnosis of pleural based lesions determined by imaging. The results may provide an alternative minimally invasive diagnostic model for pleural lesions,especially in resource-poor area.

## Methods

### Ethics statement and informed consent

The prospective study had been carried out in accordance with the Declaration of Helsinki on ethical principles for the use of human specimens for research. Each patient received written informed consent prior to participation. The study was approved by the Ethics Committee of Shanghai Pulmonary Hospital (approve number K18-170).

### Study design and participants

This study was conducted in a national pulmonary diseases specialized hospital admitted patients mainly from East China. The ultrasound department of Shanghai Pulmonary Hospital performs more than 10,000 ultrasound-interventional puncture biopsies per year.

The inclusion criteria were as follows: Adults (≥ 18 years of age) admitted to the hospital with pleural lesions (with or without pleural effusion) based on chest CT and detectable by chest ultrasound. The pleural lesions may appeared as a pleural thickening or pleural nodularity [12]. The minimal cut-off for the thickness size of the pleural based lesions was ≥ 10 mm which was visible and suitable for obtained biopsy [13]. All patients had indication for CEUS guided biopsy, which were determined by clinical specialists and sonographer, with the prior sputum or tracheoscopic results were not sufficient to eatablish a definite diagnosis.

The exclusion criteria were: a definite diagnosis was obtained by means other than pleural effusion and pleural biopsy (e.g., sputum, tracheoscopy); presence of biopsy contraindications (low platelet count, other risks such as coagulation and bleeding, clinical instability or unsafe location of biopsy); elderly patients or patients with serious systemic diseases who could not tolerate the pleural biopsy; serum HIV positive; the patients refused a pleural biopsy; pleural lesions judged by the sonographer to be unsuitable for biopsy.

When clinically necessary and safe, we performed CEUS-guided pleural biopsy. Pleural tissue was evaluated by histopathology and microbiological tests.

Patients with pleural effusion received ultrasound-guided pleural catheter drainage, and pleural effusion was tested accordingly, including routine pleural effusion examination (biochemical and ADA), microbiological and cytological tests.

### Diagnostic criteria and establishment of the final diagnosis

Diagnosis of pleural TB: We used a composite reference standard (CRS) as the diagnostic standard. CRS included both definite and possible cases. Definite cases: microbiological positive by either MTB culture or Xpert MTB/RIF(Xpert) positive on pleural effusion or pleural biopsy specimens; Possible cases: patients with TB imaging characteristics and clinical manifestations or positive TB immunological test, or histopathology confirmed as tuberculosis lesions on pleural biopsy, or biochemical examination of pleural effusion suggested TB and patients were responsive to anti-TB treatment [14].

Diagnosis of other pleural infectious diseases: Specific pathogens were identified in pleural effusion or pleural biopsy by microbiological, molecular biological or histopathological staining and targeted antibiotic treatment was effective.

Diagnosis of malignant pleural lesions: malignancy confirmed by cytological or histological examination on pleural effusion or pleural biopsy.

All patients were treated and followed up for at least 6 months. Thoracoscopy was performed on patients with undefinite diagnosis through all above examinations. A nonspecific pleurisy must be established in those with no clinical or radiological progression during follow-up [5].

### Instruments and methods

Ultrasound instrument LOGIQ E9 ultrasonic diagnostic instrument from GE, USA, with convex array probe, frequency 1–6 MHz. The instrument has harmonic contrast imaging function with low mechanical index (MI).

For patients with pleural effusion, pleural fluid was collected by closed thoracic drainage guided by ultrasound. CEUS was then used to find the target biopsy site.

*CEUS:* Conventional ultrasound was used to observe and record the size, shape, boundary, internal echo and periphery of the lesions. All patients signed the informed consent before the angiography. The instrument MI was adjusted to 0.10 and the contrast gain was 20 DB. 1.5 mL SonoVue ultrasound contrast agent (Bracco, Italy) was injected through the elbow vein. The dual-contrast interface was adopted. At the same time of the injection of contrast agent, the ultrasonic instrument was started with a built-in chronograph for 3 min continuous observation, and the images were stored and analyzed.

**Fig. 1:**
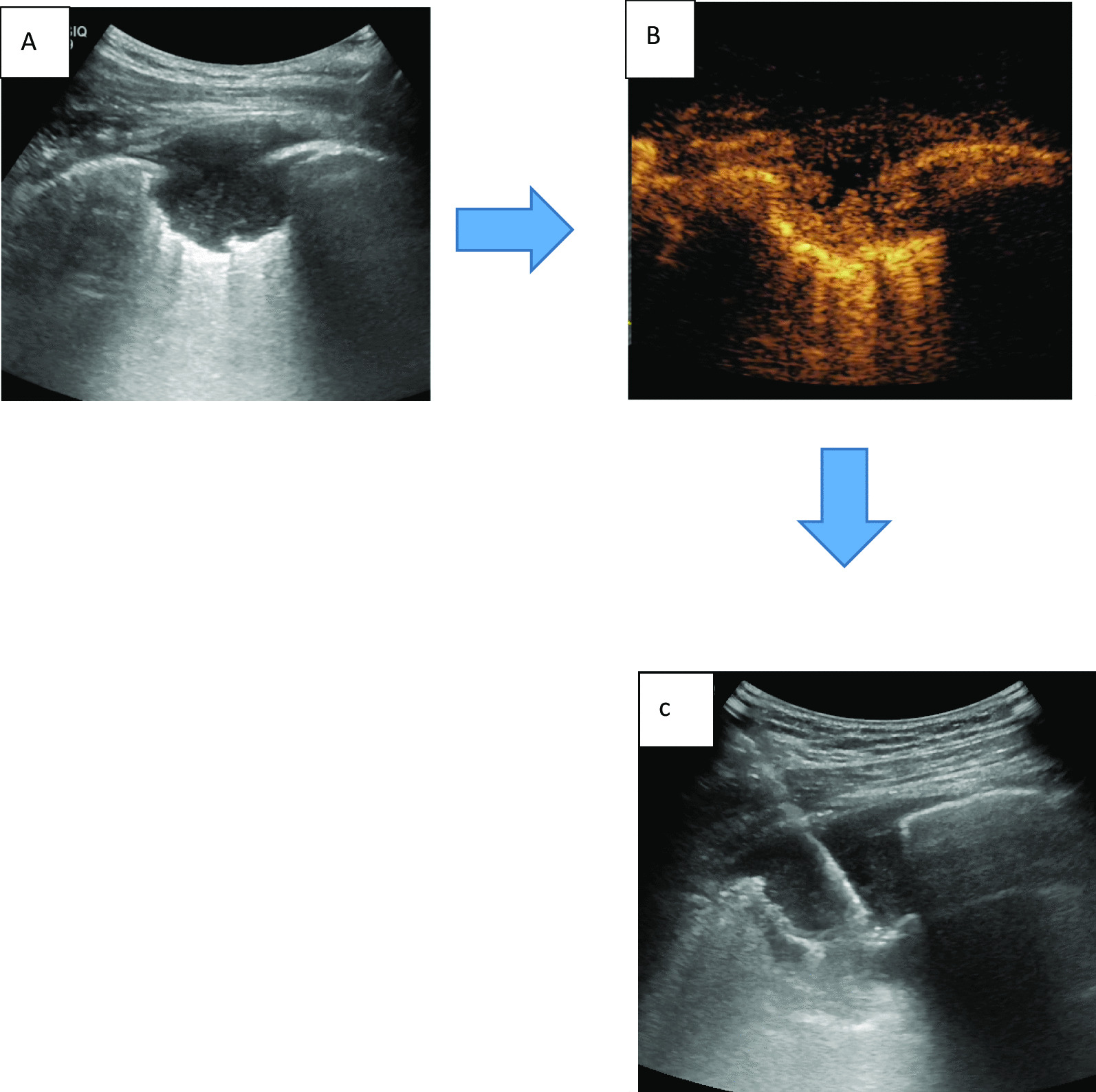
CEUS imaging of necrosis and CEUS guided biopsy. **A** conventional ultrasound showed the pleural lesion border discernible and internal echo evener with no obvious necrosis area. **B** After CEUS, the lesion center performanced as contrast agents no enhancement area, thus the necrotic area could be shown clearly seen. **C** After CEUS, ultrasound-guided percutaneous biopsy was performed, and the biopsy needle was inserted into the posterior area of the lesion

**Fig. 2:**
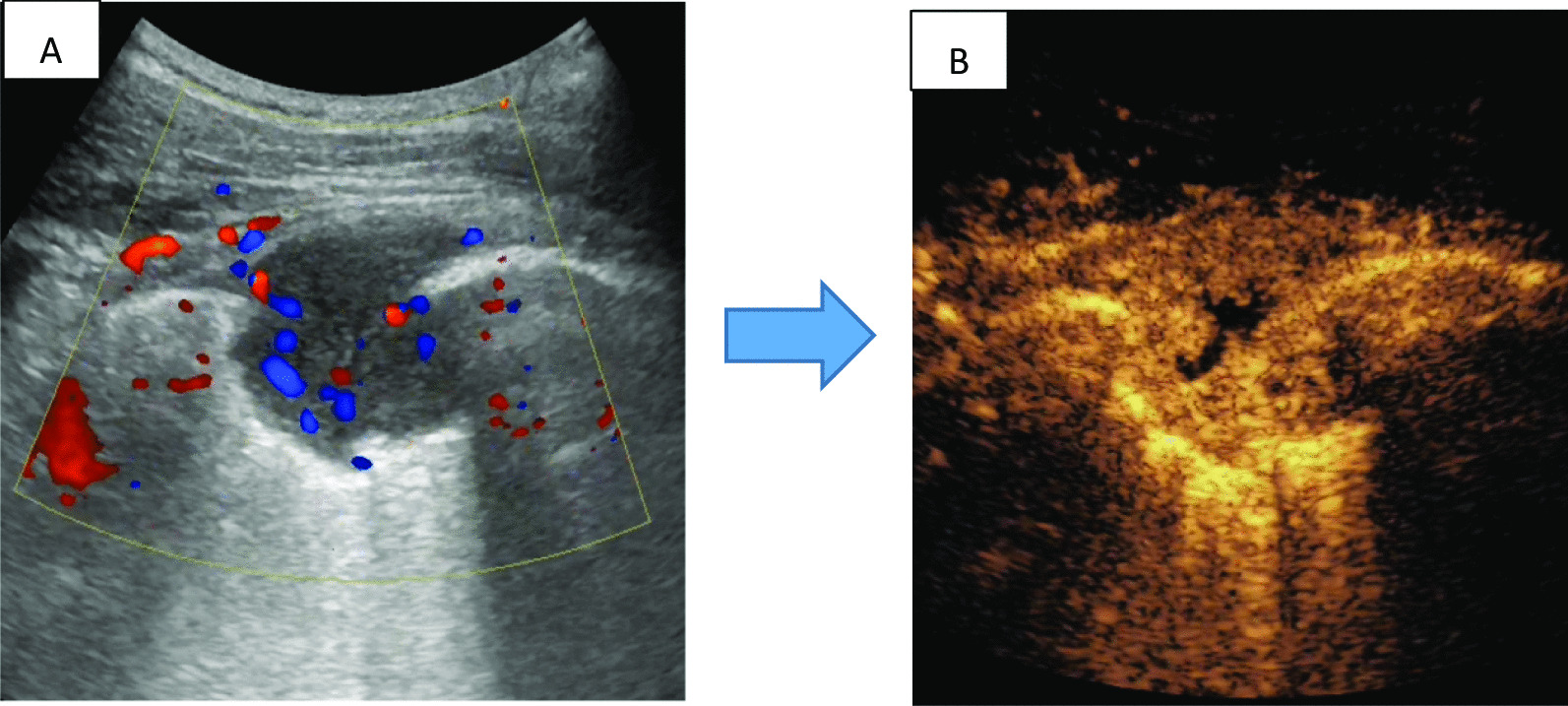
CEUS imaging of tumor blood vessels. **A** color doppler flow imaging (CDFI): article point and line sample blood flow signals within lesions. **B** significant enhancement was observed around the lesion after CEUS, suggesting rich blood supply, small unenhanced areas seen within the center

CEUS-guided biopsy: ultrasound-guided biopsy was performed immediately after CEUS. Before the biopsy, two observers evaluated the enhancement pattern. They reached a consensus to identify the viable biopsy target areas. 2% lidocaine hydrochloride was subjected to local layer. When the needle tip reached the leading edge of the enhancement area of the lesion 2–3 mm, the max-core automatic biopsy needle (model 18 G × 10 cm or 16 G × 10 cm, BD Company, USA) was excited to complete a biopsy. Repeat the operation to obtain 3–4 more complete tissue strips (Figs. 1, 2).

### Microbiological tests

The pleural tissue/pleural effusion was finely ground and suspended in 1 ml sterile saline, the pleural fluid was centrifuged (3000RPM, 15 min), the supernatant was discarded, and the precipitation solution was used for further microbiological tests (Bacteria/fungi culture), all procedures were performed according to the protocols.

BACTECTM MGIT 960 culture (MGIT 960, BD, USA) and Xpert were used for identification of mycobacterium, including mycobacterium tuberculosis (MTB) and non-Mycobacterium tuberculosis (NTM).

### Pathological examination

The biopsy specimens were fixed with 10% neutral formalin. All formalin fixation and paraffin embedding (FFPE) sections stained with hematoxylin-eosin (HE) were observed by two experienced pathologists. Specific stain and immunohistochemistry (IHC) were performed if needed.

### Complications

Complications were recorded and classified into four categories: no, self-limited pneumothorax or bleeding, pneumothorax or bleeding requiring a chest tube and hospitalization, and other major complications (death, prolonged hospitalization, etc.).

### Statistical analysis

All datas were set up in Excel 2010 and analyzed statistically with SPSS 20.0. The measurement data was compared by Student's t test. Enumeration data was compared by chi-square test. McNemar χ^2^ test was used to compare the diagnostic yield of CEUS guided biopsy specimens and pleural effusion for pleural lesions combined pleural effusion. A *p*-value of < 0.05 was considered statistically significant.

## Result

### Clinical characteristics of participants

A total of 472 patients were prospectively included from Nov 1, 2018 to Oct 31, 2019, 12 cases were excluded due to incomplete clinical data and default of follow-up. All clinical data were shown in Table 1.

**Table 1:** Clinical characteristics of patients enrolled with pleural lesions

Clinical characteristics	Infection (n = 313)	Non-infection (n = 147)	*P*
Median age, years (range)	31(16, 80)	44.6 (24,78)	0.01*
Sex n (%)			
Male	215(68.69)	80( 54.42)	0.03*
Clinical signs and symptoms n (%)			
Fever	309 (98.72)	12(8.16)	< 0.01*
Emaciation	255 (81.47)	112 (76.19)	1.42
Chest pain	190 (60.70)	120 (81.63)	< 0.01*
Chest CT imaging n (%)			
Pleural lesion	313 (100.00)	147 (100.00)	5.76
Pleural lesion with pleural effusion	240 (67.43)	90 (61.22)	1.09
CEUS performance n (%)			
Necrosis n (%)	260 (79.51)	72(54.13)	< 0.01*
Obvious peripheral vessels n (%)	146 (44.65)	109(81.95)	< 0.01*

^*^Indicated significant differences between two groups

**Fig. 3:**
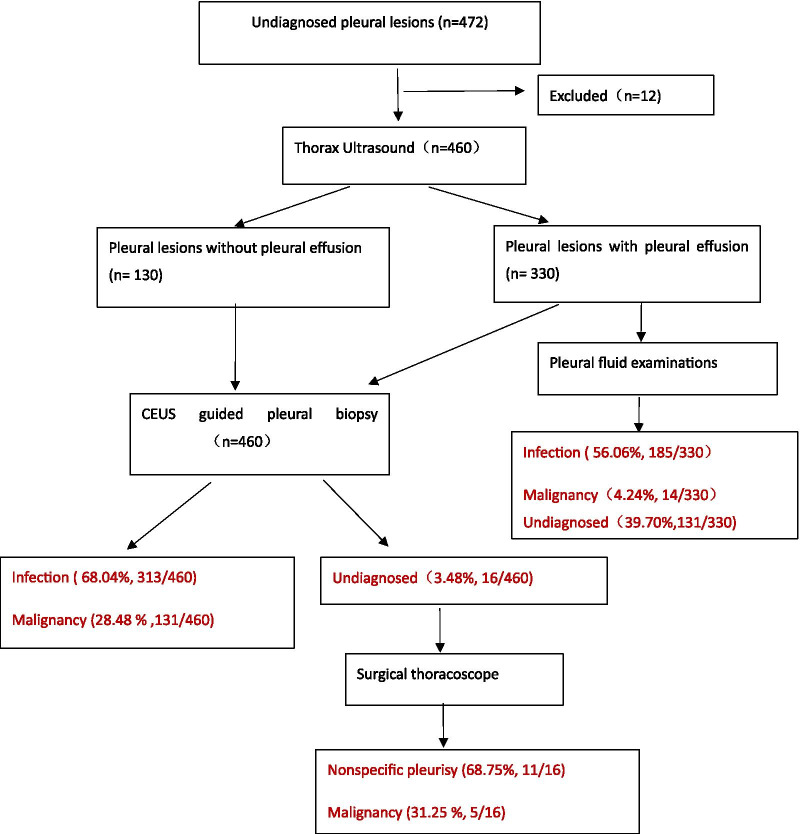
Flow diagram

Prior conventional ultrasound showed intra-lesion necrosis in 32.17% (148/460) cases and peripheral obvious vessels in 21.30% (98/460) cases, while CEUS presented intra-lesion necrosis in 72.17% (332/460) cases and obvious peripheral vessels in 55.43% (255/460) cases of the 460 cases enrolled, Both of which showed significant differences (*p* < 0.05). Intra-lesion necrosis was more likely to occur in infectious lesions ( *p* < 0.05) and intra-lesion obvious vessels were more likely to occur in non-infectious lesions (*p* < 0.05) (Figs. 1, 2, Table 1). The flow diagram was shown in Fig. 3.

### Overall diagnostic efficiency of CEUS-guided pleural biopsy sampling for pleural lesions

Among 460 cases enrolled, 444 cases obtained diagnosis through CEUS-guided biopsy directly, including 313 cases of infectious disease (280 cases of pleural TB, 10 case of NTM, 23 cases of empyema) and 131 cases of non-infectious disease (129 cases of primary/metastatic pleural malignancy, 2 cases of mesothelioma), 16 cases with undefined diagnosis were underwent thoracoscopy, then 100% (16/16) of them got the definite diagnosis of all non-infectious disease (5 cases of mesothelioma, 11 cases of nonspecific pleurisy). Therefore, thoracoscopy identified 5 additional malignant lesions on the base of CEUS-guided biopsy did.

It was worth mentioned that among the 16 cases with undefined diagnosis through CEUS guided biopsy, histopathology indicated malignant lesions in 3 cases, but the specific pathological type could not be determined, which were finally diagnosed as pleural mesothelioma through thoracoscopic surgery sampling.

In 11 cases with a final diagnosis of nonspecific pleurisy, CEUS-guided biopsies obtained the same results as thoracoscopy sampling, and all of these patients were followed up for six months with stable clinical and radiological results.

Thus, we summarized the overall diagnostic accuracy of CEUS-guided pleural biopsy was 98.91% (455/460): 100% (313/313) of infectious pleural lesions and 96.60% (142/147) of non-infectious pleural lesions.

Furthermore, in 330 cases of pleural lesions combined pleural effusion, CEUS-guided biopsy increased the diagnostic yield from 60.30% (199/330) to 98.36% (325/330, *p* < 0.05) in all cases, from 15.56% (14/90) to % 94.44% (85/90) in malignant pleural lesions (*p* < 0.01) and from 77.08% (185/240) to 100% (240/240, *p* < 0.05) in infectious pleural lesions.

### Diagnostic efficiency of CEUS-guided pleural biopsy sampling in infectious lesions

313 cases of infectious pleural lesions (280 cases of pleural tuberculosis, 10 case of NTM, 23 cases of empyema) were diagnosed through CEUS-guided biopsy. The diagnostic yield of CEUS-guided biopsy in infectious pleural lesions was 100% (313/313), meanwhile the microbiological detection rate was 71.88% (225/313).

In 240 cases with pleural lesions and pleural effusion (210 cases of pleural tuberculosis, 7 case of NTM, 23 cases of empyema), CEUS-guided biopsy obtained microbiological diagnosis in 185 cases (77.08%, 185/240), which showed significant higher than that of pleural effusion (12.08%, 29/240, *p* < 0.01). (Table 2).

**Table 2:** Microbiological detection rate of infectious pleural lesions with pleural effusion (n, %)

n = 240	Pleural tuberculosis n = 210	NTM n = 7	Empyema n = 23
Pleural effusion	25 (11.90%)	1(14.29)	3(13.04)
Pleural biopsy	159 (75.71%)	6(85.71)	20(86.96)
*P*	*p* < 0.01	*p* < 0.01	*p* < 0.01

In 280 cases of pleural tuberculosis, 100% (280/280) were diagnosed through CEUS-guided biopsy using CRS as the reference standard. The microbiological detection rate of CEUS-guided biopsy in pleural tuberculosis was 69.64% (195/280). In 210 cases pleural tuberculosis combined pleural effusion, CEUS-guided biopsy increased the diagnostic yield from 95.24% (200/210) to 100% (210/210, *P* 0.78) when using the CRS as the reference standard, meanwhile increased the microbiological detection rate from 11.90% (25/210) to 85.24% (179/210, *p* < 0.01).

### Diagnostic efficiency of CEUS-guided pleural biopsy sampling in non-infectious lesions

In 147 cases with non-infectious pleural lesions, 142 cases (129 of primary/metastatic pleural malignancy, 2 of mesothelioma, 11 cases of nonspecific pleurisy) were diagnosed through CEUS-guided biopsy. The diagnostic yield of CEUS-guided biopsy in non-infectious pleural lesions were 96.60% (142/147).

In 136 cases of malignant lesions, 131 cases (129 of primary/metastatic pleural malignancy, 2 of mesothelioma) were diagnosed through CEUS-guided biopsy. The diagnostic yield of CEUS-guided biopsy in malignant lesions were 96.32% (131/136).

In 90 cases non-infectious pleural lesions with pleural effusion, CEUS-guided biopsy obtained histopathological diagnosis in 94.44% (85/90) cases, which showed significant higher than that of pleural effusion (15.56%, 14/90, *p* <  0.01) (Table 3).

**Table 3:** Histopathologic diagnostic rate of non-infectious pleural lesions with pleural effusion (n, %)

n = 90	Pleural malignancy n = 72	Mesothelioma n = 7	Nonspecific pleurisy n = 11
Pleural effusion	14 (19.44)	0 (0.00)	0 (0.00)
Pleural biopsy	58 (100.00)	2 (28.57)	11 (100.00)
*P*	*p* < 0.01	*p* < 0.01	*p* < 0.01

### Occurrence of adverse reactions

All patients underwent CEUS and USCB. During the sampling process, 23 patients experienced minor complications. The incidence of pneumothorax was 1.1% (5/460), and most of them were small pneumothorax (compression < 15%). Only 1 patient with a history of emphysema underwent closed drainage due to pneumothorax compression of 40%. The incidence of local bleeding or sputum blood was 3.91% (18/460) and all of them got improved after short-term local pressure, and no serious adverse events occurred.

## Discussion

According to the published studies, the sensitivity of thoracoscopy for pleural lesions was 90–95% [15]. However, thoracoscopy is expensive, involves general anesthesia and invasive. Previous study showed that ultrasound-guided biopsy increased the combined diagnostic yield of all  cases  from 48.0 to 90.0%, malignancies from 31.0 to 89.7% and TB from 77.8 to 88.9% [9]. Another study of ultrasound-guided biopsy showed that 94% patients had sufficient tissue for histological diagnosis, and only one false-negative biopsy (2%) was found during the median follow-up period of 16 months [16]. As a minimally invasive method, CEUS-guided biopsy was used in the present study to locate the appropriated site and obtain valuable specimens. The overall diagnostic accuracy was 98.91%: 100% in infectious pleural lesions and 96.59% in non-infectious pleural lesions. In 330 cases of pleural lesions combined pleural effusion, CEUS-guided biopsy significantly increased the diagnostic yield from 60.30% to 98.36% in all cases, from 15.56% to % 94.44% in malignant pleural lesions and from 77.08% to 100% in infectious pleural lesions. After 6 months of follow-up, no false negative cases were found. The results showed that CEUS guided biopsy should be superior to traditional ultrasound-guided biopsy, even as high as that of thoracoscopy reported [15]. After CEUS, the location and adjacency of the unenhanced and enhanced areas in the lesion can be determined. Necrotic tissue and vessel can be clearly distinguished after injection of contrast agents which were favor for biopsy and improving the quality of aspiration tissure (Figs. 1, 2). In the present study, CEUS presented internal necrosis in 72.17% and obvious peripheral vessels in 55.43% cases of the 460 cases enrolled, which suggested that CEUS may choose a more effective and safety biopsy path than conventional ultrasonic did.

Routine treatment on pleural infectious lesions focuses on appropriate antibiotic selection and adequate drainage of pleural fluid or clear up infective lesions from the chest, closed thoracic drainage is the most common method for obtaining pleural effusion [17]. Existing study had shown that microbes are more likely to be located on the surface of the pleural wall than to "float" in the pleural fluid which is known to be acidic, hypoxic and nutrient-poor [18]. Recent studies had shown the value of pleural biopsy in the diagnosis of pleural infection and  suggested its inclusion in the routine management of pleural infection [18, 19]. However, the majority of CEUS diagnostic studies are focused on malignant tumors. Our recent study showed that CEUS-guided biopsy could increase the pathogenetic detection rate of pleural TB by accurately identifying necrosis [11]. In the present study, 100% infectious pleural lesions were diagnosed by CEUS guided biopsy and 71.88% obtained the microbiological evidence. In 240 cases with pleural effusion, CEUS guided biopsy significantly improved the microbiological diagnostic rate compared with pleural fluid extraction. Previous study had shown that in areas with high TB burdens, ultrasound-guided biopsy has a diagnostic rate of nearly 90% and a low incidence of complications [5]. In the present study, 100% pleural TB were diagnosed through CEUS guided biopsy, 69.64% of them were diagnosed microbiologically. China is a country with high TB burden. There existed a particular need to identify TB or drug-resistant TB through acquisition of pathogenic bacteria. The results of the study suggested that for pleural TB, especially "dry" pleural TB, pleural biopsy guided by CEUS may provide the rapid microbiological evidence at an early stage. In addition to TB, infectious pleurisy also included bacterial empyema and NTM. The histological manifestations of these infectious pleurisies may be similar and need to be differentiated by accurate microbiological evidence. In our study, CEUS guided biopsy detected all pathogens of infectious pleurisy other than TB, guiding the accurate antibiotic treatment.

In the present study, CEUS guided biopsy confirmed 96.32% malignant lesions. Compared with pleural effusion, biopsy specimens significantly improved  the diagnostic yield (15.56% *vs* 94.44%). Only 5 cases of mesothelioma were finally confirmed by surgical thoracoscopy. Pleural mesothelioma is a malignant pleural disease difficult to be diagnosed by minimally invasive means. Even video-assisted multipoint thoracoscopic biopsies may have a certain rate of missed diagnosis [20]. In the present study, only 28.57% mesotheliomas were diagnosed by CEUS guided biopsy. However, it is worth noting that even among the 5 cases of mesothelioma with uncertain diagnosis through CEUS-guided biopsy, 3 of them showed pathologically malignant lesions.

In addition, the advantage of CEUS-guided biopsy is that the entire needle biopsy process is performed under direct vision. It monitors the movement of the lungs in real time, allowing patients to hold their breath and perform the puncture immediately to avoid injury. In the present study, the incidence of complications was only 5%, all of which were mild and relieved after short-term symptomatic treatment. Our results indicated that CEUS biopsy provides a safety diagnostic approach.

Our study had the following limitations: we did not set traditional ultrasound guided biopsy without contrast-enhanced agents as the control. Because ultrasound contrast injection could greatly improve the differentiation of necrotic and vascular areas (Figs. 1, 2), all included patients were performed by CEUS guided biopsy under safety and ethical consideration. On the other hand, existing study also shown that ultrasound contrast agent injection may have little effect on the accuracy of lung cancer diagnosis. For the accuracy of the diagnosis may be affected by the operator's experience、the number of biopsies、 the needle used for the biopsy and the experience of the pathologist, etc [21]. Secondly, all patients with indications of pleural examination were included in the study, including those who had been definitively diagnosed by pleural effusion cytology or microbiology, the actual proportion of these patients in the study is not large (only 29 in infectious pleural effusion and 14 in malignant pleural effusion). If the patients were strictly enrolled by pre-examination, most of them may also face delays in repeated tests. However,  for malignant tumors, the quality of histomathological specimens obtained by biopsy may be of better value for patients' subsequent genetic test and the need for repeated follow-up tests (compared to cytological specimens of pleural effusion or puncture). And for infectious diseases, biopsy specimens could also obtain a better microbial population.

In conclusion, by identifying necrosis and surrounding blood vessels, CEUS guided a more effective biopsy that was proved to be safe and minimally invasive. It was considered to be applied as an effective diagnostic method for patients with pleural lesions, especially in resource-poor areas.

## Data Availability

All data regarding the included participants and laboratory data during the study are available from the corresponding author by email request. The clinical study was registered at The China Clinical Trial Registry (ChiCTR, www.chictr.org.cn) with the registration number: ChiCTR2000029749.
